# Comparison of corneal thickness in patients with dry eye disease using the Pentacam rotating Scheimpflug camera and anterior segment optical coherence tomography

**DOI:** 10.1371/journal.pone.0228567

**Published:** 2020-02-03

**Authors:** Keiichi Fujimoto, Takenori Inomata, Yuichi Okumura, Nanami Iwata, Kenta Fujio, Atsuko Eguchi, Ken Nagino, Hurramhon Shokirova, Maria Karasawa, Akira Murakami

**Affiliations:** 1 Juntendo University Graduate School of Medicine, Department of Ophthalmology, Tokyo, Japan; 2 Juntendo University Faculty of Medicine, Department of Ophthalmology, Tokyo, Japan; 3 Juntendo University Faculty of Medicine, Department of Strategic Operating Room Management and Improvement, Tokyo, Japan; 4 Juntendo University Graduate School of Medicine, Department of Hospital Administration, Tokyo, Japan; Bascom Palmer Eye Institute, UNITED STATES

## Abstract

The purpose of this study was to compare central corneal thickness, thinnest corneal thickness, and the thinnest point of the cornea between Pentacam and anterior segment optical coherence tomography (ASOCT) in patients with dry eye disease (DED). This cross-sectional study included 195 participants between November 2015–June 2017. DED was diagnosed using the Asia Dry Eye Society criteria and further divided into mild and severe DED based on kerato-conjunctival vital staining. Central corneal thickness, thinnest corneal thickness, and the thinnest point of the cornea measured by Pentacam and ASOCT were compared, and Pearson’s correlation coefficients were estimated. The differences in central corneal thickness and the thinnest corneal thickness between Pentacam and ASOCT were analysed using Bland–Altman and multivariate regression analyses adjusted for age and sex. This study included 70 non-DED subjects and 52 patients with mild and 73 with severe DED. The Pentacam and ASOCT measurements of central corneal thickness and thinnest corneal thickness were strongly correlated, but the respective values were higher when measured with Pentacam. The Bland–Altman analysis revealed differences in central corneal thickness (non DED, 11.8; mild DED, 13.2; severe DED, 19.6) and in thinnest corneal thickness (non DED, 13.1; mild DED, 13.4; severe DED, 20.7). After adjusting for age and sex, the differences in central corneal thickness (β = 7.029 μm, 95%CI 2.528–11.530) and thinnest corneal thickness (β = 6.958 μm, 95%CI 0.037–13.879) were significantly increased in the severe-DED group. The distribution of the thinnest point of the cornea in the cornea’s inferior temporal quadrant between Pentacam and ASOCT deviated in severe DED (Pentacam: 90.4% vs. ASOCT: 83.6%). Clinicians should consider that there were significant differences in corneal-morphology assessment between the measurements with Pentacam and ASOCT in severe DED.

## Introduction

Central corneal thickness (CCT) is a corneal health indicator, including of metabolism and hydration status [1], and an important parameter for the characterization of corneal disease including keratoconus and Fuchs dystrophy due to the barrier and endothelial pump function of the cornea [2–4]. Accurate measurements of CCT affect the outcomes of refractive surgery, corneal transplantation, and contact-lens prescriptions [5–7]. An accurate CCT value is essential for the adjustment of intraocular pressure (IOP) in each patient with glaucoma, as CCT is a potent confounder of most tonometry techniques [8]. In addition, CCT could be an independent risk factor of glaucoma [8, 9].

The incidence of dry eye disease (DED) has been increasing with the aging society and increased digital devise usage, affecting tens of millions of people worldwide [10–12]. Many people were estimated to have undiagnosed DED with decreased quality of vision and work productivity [13]. DED was found to be comorbid with post cataract surgery and glaucoma treatment due to shared risk factors including aging and the long-term use of eye drops containing preservatives [14–16]. As contact-lens use is a risk factor of DED [13], patients with keratoconus who use hard contact lenses for suppression of the condition’s development could develop DED [17]. Therefore, the accurate assessment of CCT in DED is becoming more important. Many studies have been conducted on DED and CCT, reporting thinner CCT in patients with DED and that the measurement of CCT is useful for the follow up of DED [18–20].

The diagnosis of DED is based on multiple DED examinations including of subjective symptoms, tear film break-up time (TFBUT), tear osmolality, kerato-conjunctival vital staining, and tear volume [21, 22]. As kerato-conjunctival epithelial damage in DED varies from mild to severe, it is necessary to determine the appropriate CCT assessment method according to the kerato-conjunctival epithelial damage in DED. In addition, since severe DED causes corneal perforation, it is important to assess thinnest corneal thickness (TCT) and the thinnest point of the cornea in DED. Previous studies have reported that the thinnest point of the cornea in healthy eyes is mostly located in the inferior temporal quadrant of the cornea [4, 23, 24]. However, few studies have assessed the TCT and the thinnest point of the cornea in patients with DED [25].

There are various methods available to measure CCT. Ultrasound pachymetry (USP) has been widely used as the gold standard because of its reproducibility and portability. However, alternative methods of CCT assessment have been proposed because USP has several limitations including cornea-probe contact, the need for topical anesthesia, corneal indentation, a possible compression effect, and risk of corneal erosions [5]. Especially in the case of DED, non-contact measurement methods are preferred because the patients with DED are more susceptible to corneal epithelial damage and infection [26]. Recently, several units of non-contact tonometry and pachymetry have been developed for clinical and small-animal imaging [27]. Pentacam (OCULUS, Optikgerate GmbH, Wetzlar, Germany), a rotating Scheimpflug camera, and anterior segment optical coherence tomography (ASOCT) determine the CCT, TCT, and the thinnest point of the cornea. However, few studies have examined the differences in the measurement of corneal morphology using Pentacam and ASOCT in patients with DED [25, 28] and no study has compared them.

Therefore, this study aimed to compare the measurement of CCT, TCT, and the thinnest point of cornea using two non-contact devices, Pentacam and ASOCT, in patients with DED.

## Materials and methods

### Study design and participants

This cross-sectional retrospective study included 195 participants recruited between November 2015 and June 2017 from the Department of Ophthalmology at Juntendo University Hospital in Tokyo, Japan. The requirement for written informed consent was waived due to the retrospective observational nature of the study, and the study was carried out using the opt-out method on our hospital website. The study was approved by the Independent Ethics Committee at Juntendo University Hospital (Approval number 17–088) and adhered to the tenets of the Declaration of Helsinki.

### Exclusion criteria

We excluded patients with a history of diabetes, uveitis, glaucoma, increased IOP, eyelid disorder, ptosis, and any corneal disease including herpetic keratitis, endothelial guttae, hereditary corneal disease, contact lens wear, eye surgery, and eyelid surgery.

### Environmental conditions

The temperature of the examination room was controlled at 26°C in the summer and 24°C in the winter with 50% relative humidity, according to the Guideline for Design and Operation of Hospital HVAC Systems established by the Healthcare Engineering Association of Japan [29].

### Dry eye disease diagnosis and classification

All patients underwent a complete ophthalmic evaluation, including measuring best-corrected visual acuity, IOP, endothelial cell density (EM-3000 Specular microscope, Tomey Corporation, Nagoya, Japan), and subjective symptom assessment using the Dry Eye-Related Quality-of-Life Score (DEQS) [30]. Additionally, TFBUT, kerato-conjunctival vital staining, and Schirmer’s test I for reflex tear production in the right eye were assessed. For each patient, we evaluated TFBUT and kerato-conjunctival vital staining before performing Schirmer’s test I. We diagnosed DED and non-DED using the Asia Dry Eye Society 2016 diagnostic criteria [22], which are based on two positive items: the presence of subjective symptoms and decreased TFBUT (≤5 seconds). Then, the DED group was divided into mild DED without vital staining (score <3) and severe DED with vital staining (score ≥3) according to the van Bijsterveld grading system [31].

### Subjective symptoms and Dry Eye-Related Quality-of-Life Score

Subjective symptoms were evaluated using the DEQS questionnaire in order to assess the severity of dry eye-associated symptoms and the multifaceted effects of DED on daily life [30]. The score derived from this questionnaire is a subjective measurement of DED symptoms, where 0 indicates the best score (no symptoms) and 100 indicates the worst score (maximum symptoms). A cut-off value of DEQS >15 was used to diagnose DED [32].

### Tear film break-up time

TFBUT was measured using a fluorescein dye according to the standard method [22]. Only a small quantity of dye was administered using the wetted fluorescein strip to minimize the effect of the dye on tear volume and TFBUT. Each subject was instructed to blink three times after the dye was applied to ensure adequate mixing of the dye and tears. The time interval between the last blink and the appearance of the first dark spot on the cornea was measured with a stopwatch. The mean value of three measurements was used. A cut-off value of TFBUT ≤5 seconds was used to diagnose DED [22].

### Kerato-conjunctival vital staining

Kerato-conjunctival vital staining was graded according to the van Bijsterveld grading system [31], which divides the ocular surface into three zones: the nasal bulbar conjunctiva, the temporal bulbar conjunctiva, and the cornea. Each zone was evaluated on a scale of 0–3, with 0 indicating no staining and 3 indicating confluent staining. The maximum possible score was thus 9.

### Schirmer’s test I

Schirmer’s test I was performed without topical anesthesia after all other examinations had been completed. Schirmer’s test strips (Ayumi Pharmaceutical Co., Tokyo, Japan) were placed on the outer third of the temporal lower conjunctival fornix for 5 minutes. The strips were then removed, and the length of dampened filter paper (in mm) was recorded.

### Corneal thickness assessment

CCT, TCT, and the thinnest point of the cornea were determined with Pentacam (Oculus Optikgerate GmbH) and ASOCT (CASIA, SS1000-OCT, Tomey) as detailed previously [24, 33, 34]. The instruments were calibrated and utilized according to the manufacturer instructions. Corneal assessments using Pentacam and ASOCT were conducted before the DED examinations including TFBUT and kerato-conjunctival vital staining because it is known that the tear film after fluorescein staining affects the CCT measurement by Pentacam [25].

Briefly, Pentacam stated the measurement automatically when correct alignment with the corneal apex and focus was achieved. The noninvasive measurement process of the Pentacam requires 2 seconds, collecting 25 to 50 single captures consisted of 13,800 true elevation points while rotating around the optical axis of the eye, resulting in a Pentacam built 3-dimensional model of the entire anterior eye segment.

OCT was initially developed for the assessment of the retina [35]. OCT has improved image scanning and has enabled the noninvasive quantification of both the fine structures of the retina and of the anterior segment of the eye. The CASIA is a swept source OCT (SS-OCT) device that uses a long-wavelength light source with a central wavelength of 1,310 nm and has a speed of 30,000 axial scans per second. CASIA swept source OCT is an elevation-based topographic modality that acquires corneal shape data from above the height of the cornea. The Top-Pachy-Map protocol was implemented in CASIA to measure corneal thickness and topography with 16 radial cross-sections and total scan time 0.3 seconds.

### Statistical analyses

Participant characteristics are presented by DED subgroup (non DED, mild DED, and severe DED). We conducted one-way analysis of variance with Bonferroni’s multiple comparisons to compare the mean values between multiple groups and chi-square tests for categorical variables among the groups. The CCT and TCT measured by Pentacam and ASOCT were compared by paired t test. Pearson’s correlation coefficients between CCT measured with Pentacam and CCT measured with ASOCT were calculated for each group separately. The same was performed for TCT. Bland–Altman analysis [36] was conducted to provide indication of the systematic random error and the heteroscedasticity of the data and the 95% limits of the agreement between Pentacam and ASOCT. We compared the differences in CCT and TCT between Pentacam and ASOCT by regression analysis adjusted for age and sex. Data are presented as mean ± standard deviation (SD), standard error (SE), or proportion (%). Statistical analyses were performed using STATA version 15 (Stata Corp, College Station, TX) and GraphPad Prism version 7.0 (GraphPad Software, San Diego, CA). *P*<0.05 was considered significant.

## Results

### Participant characteristics

**Table 1** shows the general characteristics of the study participants. All subjects responded to the questionnaires, completed the examination, and were eligible for the study. The mean age was 63.2±13.1 years and 77.4% of the participants were female. One hundred and ninety-five participants were divided into non-DED (35.9%, n = 70), mild-DED (26.7%, n = 52), and severe-DED (37.4%, n = 73) groups. The severe-DED group included younger and a higher proportion of female participants. The DED examination results, including the DEQS score and TFBUT, were worse in the DED groups than in the non-DED group. The kerato-conjunctival vital staining score was increased in the severe-DED compared to the non-DED group, whereas it was decreased in the mild-DED group compared to the non-DED group because of the group classification in this study.

**Table 1:** Characteristics of study participants.

	Non DED	Mild DED	Severe DED	*P* value	Total
	n = 70	n = 52	n = 73		n = 195
Age, years ± SD	65.8 ± 12.4	64.6 ± 12.9	59.8 ± 13.5	*0.015	63.2 ± 13.1
Female, n (%)	43 (61.4)	42 (80.8)	66 (90.4)	***< 0.001	151 (77.4)
BCVA, LogMAR ± SD	0.03 ± 0.15	0.04 ± 0.17	0.01 ± 0.16	0.719	0.02 ± 0.16
IOP (mmHg) ± SD	14.1 ± 3.0	14.4 ± 2.7	13.3 ± 2.8	0.155	13.9 ± 2.9
ECD, cells/mm^2^ ± SD	2,663 ± 280	2,641 ± 359	2,611 ± 357	0.870	2,627 ± 331
DEQS score ± SD	13.3 ± 18.0	39.7 ± 19.9	41.7 ± 18.3	***< 0.001	31.0 ± 22.8
TFBUT, seconds ± SD	3.7 ± 3.2	2.3 ± 1.3	1.9 ± 1.1	***< 0.001	2.6 ± 2.3
Kerato-conjunctival vital staining score ± SD	2.4 ± 2.3	0.8 ± 0.7	4.0 ± 1.4	***< 0.001	2.6 ± 2.1
Schirmer 1, mm ± SD	6.9 ± 5.7	6.2 ± 5.2	7.0 ± 7.6	0.737	6.7 ± 6.3

*P* values were determined with Student’s t-tests and one-way analysis of variance for continuous variables and the chi-square test for categorical variables. DED: dry eye disease, BCVA: best-corrected visual acuity, IOP: intraocular pressure, ECD: endothelial cell density, DEQS; the Dry Eye-Related Quality-of-Life Score, TFBUT: tear film break-up time. Data are considered statistically significant at *P<0.05, ***P<0.001.

### Corneal thickness assessment in the patients with DED using Pentacam and ASOCT

**Fig 1:**
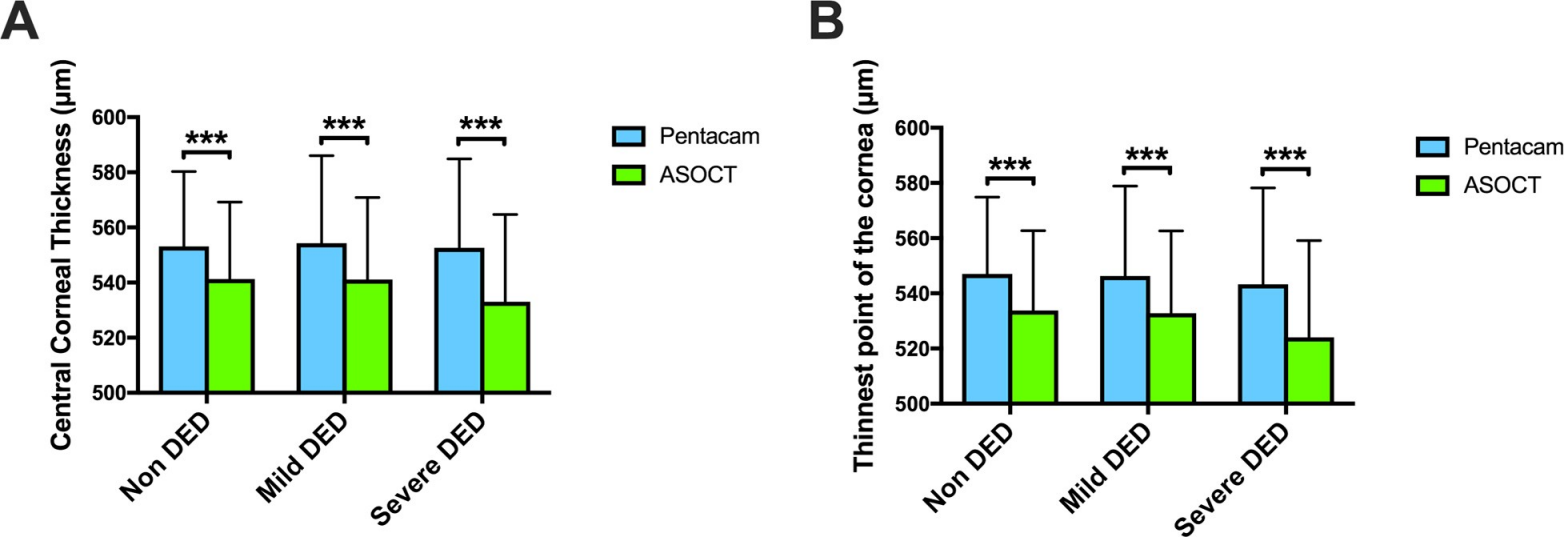
Comparison of corneal thickness assessment between Pentacam and ASOCT in patients with Dry Eye Disease. (**A**) The central corneal thickness and (**B**) the thinnest points of the cornea were compared between Pentacam and ASOCT according to the subgroups. Data are considered statistically significant at ***P < 0.001. DED: dry eye disease, ASOCT: anterior segment optical coherence tomography.

**Fig 2:**
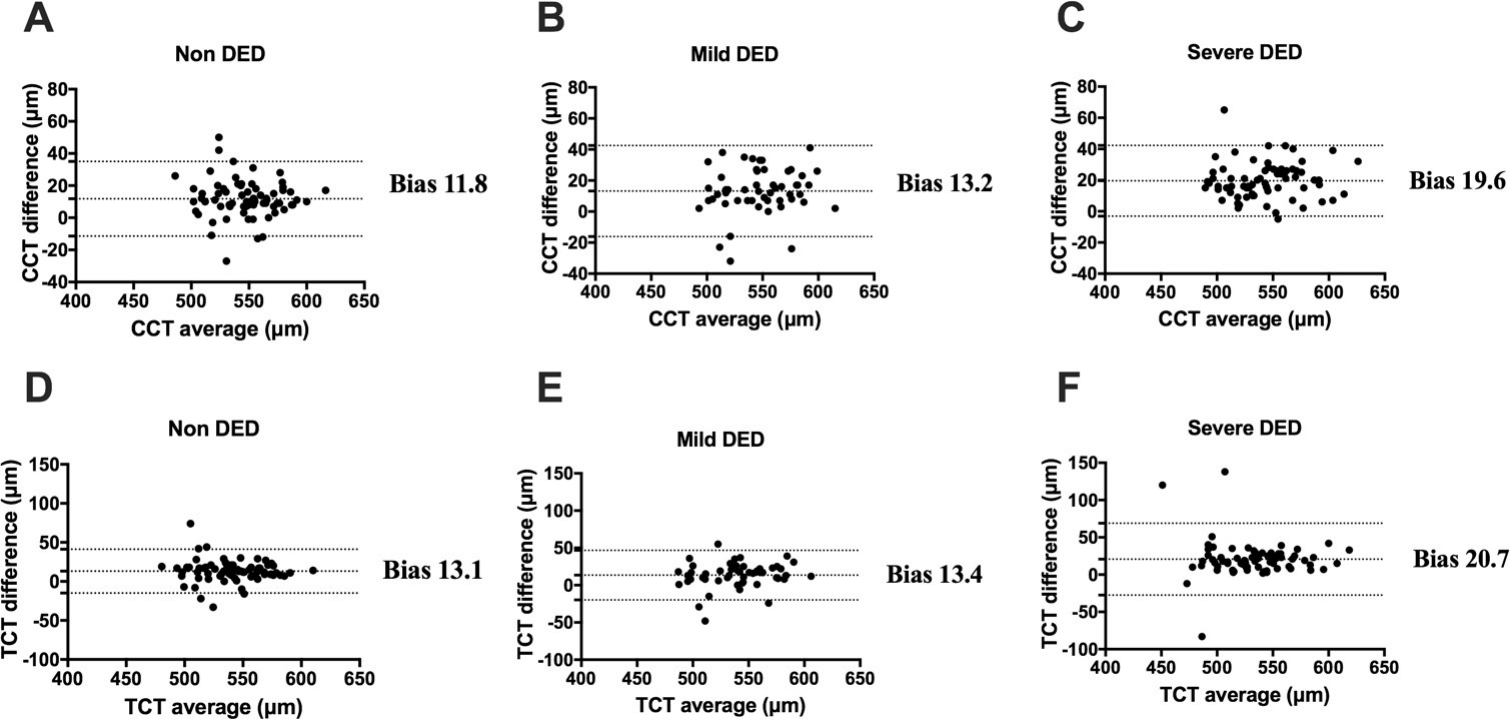
Bland–Altman plot for central corneal thickness and thinnest point of the cornea for Pentacam and ASOCT. The x-axis indicates the average of the thickness between the Pentacam and ASOCT measurements, and the y-axis indicates the difference between Pentacam and ASOCT (Pentacam-ASOCT). The central line indicates the mean difference (bias) between the normalized thickness from the two devices, whereas the superior and inferior lines depict the intervals that include 95.6% of all differences. (**A**)–(**C**) show the bias for CCT and (**D**)–(**E**) for TCT. ASOCT: anterior segment optical coherence tomography, CCT: central corneal thickness, TCT: thinnest corneal thickness.

**Fig 1** shows the assessment of corneal thickness, including CCT and TCT, using Pentacam and ASOCT according to the subgroups. **Fig 1A** shows that the mean CCTs (±SD), measured by Pentacam and ASOCT, were in non DED (553.1±27.2 μm vs. 541.3±27.9 μm, *P*<0.001), mild DED (554.3±31.7μm vs. 541.1±29.8 μm, *P*<0.001), and severe DED (552.6±32.3 μm vs. 533.0±31.7 μm, *P*<0.001). **Fig 2B** shows that the mean TCTs (±SD), measured by Pentacam and ASOCT, were in non DED (547.0±27.9 μm vs. 533.8±28.9 μm, *P*<0.001), mild DED (546.3±32.6 μm vs. 532.8±29.8 μm, *P*<0.001), and severe DED (543.3±34.9 μm vs. 524.0±35.1 μm, *P*<0.001). **Table 2** shows the correlation coefficients between the Pentacam and ASOCT measurements per group for CCT (**Table 2A**) and TCT (**Table 2B**). There were strong positive correlations between the Pentacam and ASOCT measurements for both CCT and TCT in each subgroup.

**Table 2:** Correlation coefficients between the Pentacam and ASOCT measurements for CCT and TCT per Group.

A. CCT (Pentacam vs. ASOCT)	r	*P* value
Non DED	0.9081	***< 0.001
Mild DED	0.8835	***< 0.001
Severe DED	0.9348	***< 0.001
B. TCT (Pentacam vs. ASOCT)	r	*P* value
Non DED	0.8735	***< 0.001
Mild DED	0.8561	***< 0.001
Severe DED	0.7634	***< 0.001

CCT: central corneal thickness, TCT: thinnest corneal thickness, ASOCT: anterior segment optical coherence tomography, DED: dry eye disease. Data are considered statistically significant at ***P<0.001.

### Bias of CCT and TCT between Pentacam and ASOCT

**Fig 2** shows the results of the Bland–Altman analysis for corneal thickness, including CCT (**Fig 2A–2C**) and TCT (**Fig 2D–2F**), between Pentacam and ASOCT. The CCT differences (bias ±SD (limits of agreement)) were 11.8±11.9 (-11.42–35.02) in the non-DED (**Fig 2A**), 13.2±15.0 (-16.14–42.52) in the mild-DED (**Fig 2B**), and 19.6±11.6 (-3.13–42.25) in the severe-DED (**Fig 2C)** group. The TCT differences (bias) were 13.1±14.2 (-14.94–41.19) in the non-DED (**Fig 2D)**, 13.4±17.0 (-19.81–46.69) in the mild-DED (**Fig 2E)**, and 20.7±24.6 (-27.49–68.92) in the severe-DED (**Fig 2F)** group.

### Adjusted differences in CCT and TCT by regression analysis

**Table 3** shows the adjusted differences in CCT and TCT using Pentacam and ASOCT based on the groups. After adjusting for age and sex in the multivariable regression analysis, the differences in CCT (**Table 3A**, β = 7.029 μm, 95%CI 2.528–11.530, *P* = 0.002) and TCT (**Table 3B**, β = 6.958 μm, 95%CI 0.037–13.879, *P* = 0.049) were significantly increased in the severe-DED group compared to the non-DED group.

**Table 3:** Adjusted CCT and TCT differences between Pentacam and ASOCT.

A. CCT	Coefficient	SE	*P* value	95%CI
Non DED	1 (reference)	–	–	–
Mild DED	0.885	2.367	0.709	-3.783–5.554
Severe DED	7.029	2.282	**0.002	2.528–11.530
Age	0.007	0.071	0.927	-0.134–0.148
Sex	2.663	2.300	0.248	-1.874–7.200
B. TCT	Coefficient	SE	*P* value	95%CI
Non DED	1 (reference)	–	–	–
Mild DED	-0.153	3.639	0.966	-7.331–7.024
Severe DED	6.958	3.509	*0.049	0.037–13.879
Age	0.018	0.110	0.872	-0.199–0.235
Sex	2.529	3.536	0.475	-0.446–9.504

DED: dry eye disease, SE: standard error, 95%CI: 95% confidence interval. Data are considered statistically significant at **P<0.01 and *P<0.05.

### Differences in the location of the thinnest point of the cornea by Pentacam and ASOCT

**Fig 3:**
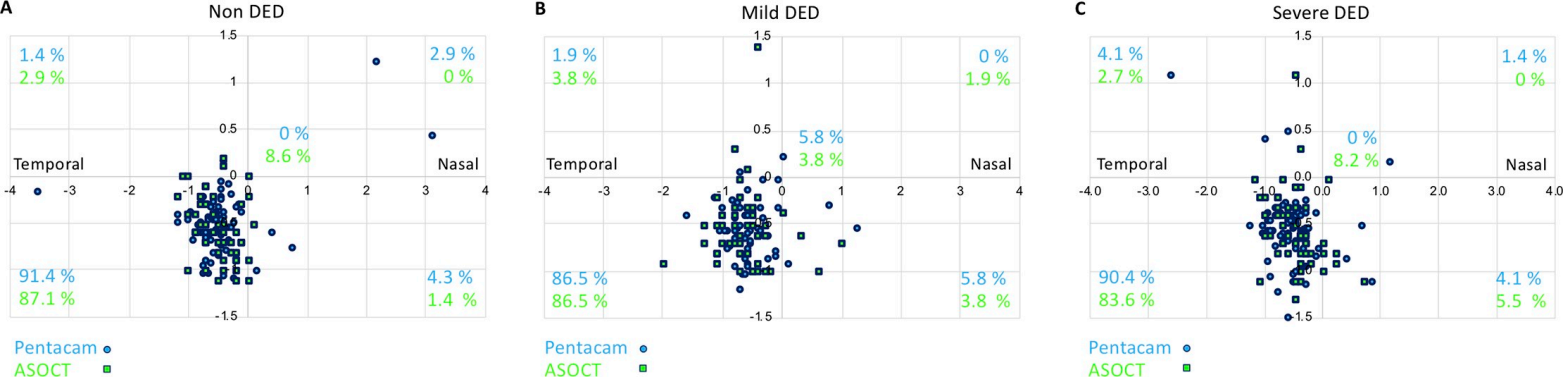
Location of the thinnest point of the cornea as measured by Pentacam and ASOCT. The location of the thinnest point of the cornea was mapped from its quadrant location using Pentacam and ASOCT based on the (**A**) non-DED group, (**B**) mild-DED group, and (**C**) severe-DED group. DED: dry eye disease, ASOCT: anterior segment optical coherence tomography.

**Fig 3** shows the location of the thinnest point of the cornea measured by Pentacam and ASOCT. The distribution of the thinnest point in the inferior temporal quadrant of the cornea was in non DED (**Fig 3A**, Pentacam: 91.4% vs. ASOCT: 87.1%, difference: 4.3%), mild DED (**Fig 3B**, 86.5% vs. 86.5%, 0%), and severe DED (**Fig 3C**, 90.4% vs. 83.6%, 6.8%).

## Discussion

Accurate assessment of corneal thickness is important not only for monitoring corneal homeostasis but also for refractive surgery and IOP management in patients with glaucoma. The measurement of corneal thickness has changed from the invasive method used in the past, i.e., USP, to noninvasive methods including assessment with Pentacam and ASOCT. These devices rely on different underlying technical principles to assess corneal morphology. Therefore, it is important to assess the accuracy of these examinations because DED is accompanied by kerato-conjunctival epithelial damage. This study showed that there is a difference in CCT, TCT, and the distribution of the thinnest point of cornea when measured with Pentacam or ASOCT in patients with severe DED.

A previous study reported that Pentacam and ASOCT can substitute USP for CCT measurement [37]. Although the reliability of Pentacam and ASOCT has been confirmed in DED [25, 28], no study has compared Pentacam and ASCOT in patients with DED. This study showed that there was a strong positive correlation between the Pentacam and ASOCT measurements of CCT in each subgroup (**Table 2**); however, CCT was significantly higher when measured with Pentacam than when measured with ASOCT in all subgroups (**Fig 1**). These results were in accordance with those of previous studies reporting that CCT measured with Pentacam was thicker compared to that measured with ASOCT in healthy corneas [5, 37, 38]. Our Bland–Altman analysis showed that the Pentacam and ASOCT measurements tended to have good agreement for the CCT measurements in the non-DED and mild-DED groups; however, Pentacam provided thicker CCT compared to ASOCT in the severe-DED group (**Fig 2**). These differences were also confirmed by multivariable regression analysis adjusted for age and sex, indicating that clinicians should be aware of the differences in CCT measurements between Pentacam and ASOCT in patients with severe DED with kerato-conjunctival epithelial damage. The reason for the increased CCT difference between Pentacam and ASOCT in severe dry eye is that Pentacam is less susceptible to tears [39] and ASOCT is possibly influenced by corneal surface heterogeneity due to decreased tear production and increased kerato-conjunctival epithelial disorder in severe DED.

This is the first study to report that there was a strong positive correlation between the Pentacam and ASCOT measurements of TCT in each subgroup (**Table 2**); however, the TCT was significantly higher when measured with Pentacam than when measured with ASOCT in all subgroups (**Fig 1**). The differences in TCT measurements between Pentacam and ASOCT were increased in the severe-DED group based on the Bland–Altman analysis as shown in **Fig 2** and as verified by multivariable regression analysis adjusted for age and sex. Our study showed that the location of the thinnest point of the cornea in patients with DED was mostly located in the inferior temporal quadrant of the cornea, as also found in healthy eyes [4, 23, 24]; however, the distribution at which the thinnest point was located in the inferior temporal quadrant of the cornea deviated in the severe-DED group between Pentacam and ASOCT (**Fig 3**). Therefore, it is important to be aware that errors may occur between Pentacam and ASOCT when examining the thinnest point in cases with corneal thinning including in patients with infectious corneal disease, autoimmune disease, severe DED, and chronic graft-versus-host disease. Furthermore, as the corneal epithelial damage in DED was determined based on the subtype of DED [40], further investigation for the evaluation based on DED subtype between Pentacam and ASOCT is needed.

This study has a few limitations. First, this study may have involved selection bias due to the single center design in a university hospital. Another limitation is that CCT and TCT were only evaluated for the comparison of the devices in patients with DED; however, evaluation of the entire cornea is needed because DED affects various parts of the cornea.

## Conclusions

This study showed that there were significant differences in the CCT, TCT, and the distribution of the thinnest point of the cornea between Pentacam and ASOCT in severe DED. Clinicians should be aware that in severe DED, there may be differences in corneal assessment, especially in CCT, TCT, and the distribution of the thinnest point of the cornea, according to the measurement method used.
